# The Well-Developed Mucosal Immune Systems of Birds and Mammals Allow for Similar Approaches of Mucosal Vaccination in Both Types of Animals

**DOI:** 10.3389/fnut.2018.00060

**Published:** 2018-07-12

**Authors:** Tomonori Nochi, Christine A. Jansen, Masaaki Toyomizu, Willem van Eden

**Affiliations:** ^1^International Education and Research Center for Food and Agricultural Immunology, Graduate School of Agricultural Science, Tohoku University, Miyagi, Japan; ^2^International Research and Development Center for Mucosal Vaccine, The Institute of Medical Science, The University of Tokyo, Tokyo, Japan; ^3^Department of Infectious Diseases and Immunology, Faculty of Veterinary Medicine, Utrecht University, Utrecht, Netherlands

**Keywords:** mucosal immune system, mucosal vaccine, nutritional supplementation, mammals, birds

## Abstract

The mucosal immune system is a compartmentalized part of the immune system that provides local immunity in the mucosa of the respiratory, gastrointestinal, and digestive tracts. It possesses secondary lymphoid tissues, which contain immune cells, such as T, B, and dendritic cells. Once the cells of the mucosal immune system are stimulated by luminal antigens, including microorganisms, they infiltrate into diffuse areas of mucosal tissues (e.g., respiratory mucosa and lamina propria of intestinal villi) and exhibit immune effector functions. Inducing the antigen-specific immune responses in mucosal tissues by mucosal vaccination would be an ideal strategy for not only humans, but also mammals and birds, to protect against infectious diseases occurring in mucosal tissues (e.g., pneumonia and diarrhea). Infectious diseases cause huge economic losses in agriculture, such as livestock and poultry industries. Since most infectious diseases occur in mucosal tissues, vaccines that are capable of inducing immune responses in mucosal tissues are in high need. In this review, we discuss the current understanding of mucosal immunity in mammals and birds, and recent progress in the development of mucosal vaccines.

## The mucosal immune system in mammals and birds

**Figure 1:**
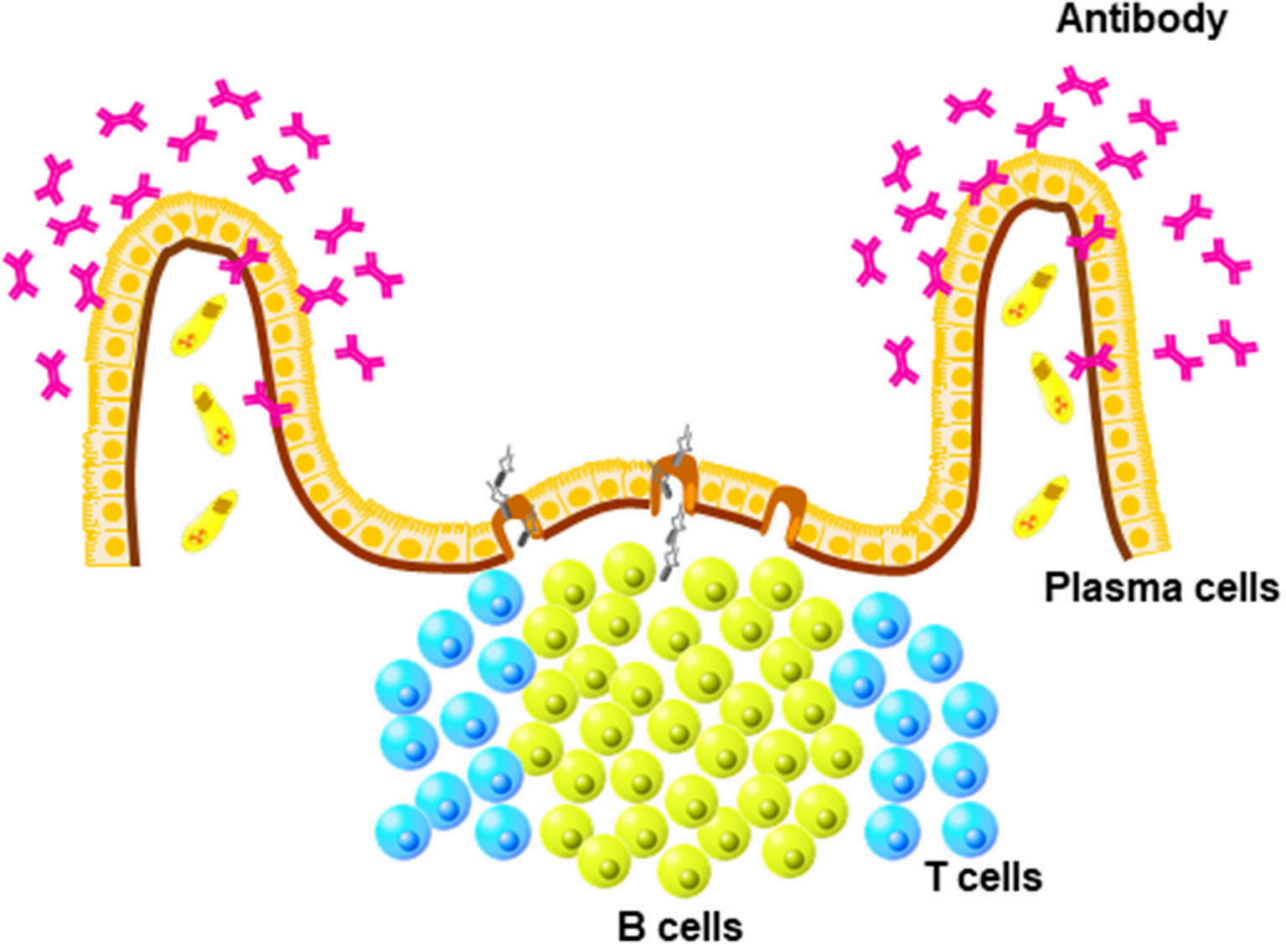
Unique structure of mucosa-associated lymphoid (MALTs). MALTs contain mature B cells that differentiate into antibody-producing plasma cells present in diffuse areas of mucosal tissues, such as lamia propria.

The mucosa-associated lymphoid tissues, which are lymphoid structures in the mucosal tissues, form the first line of defense against pathogens that enter the body through the mucosal surfaces lining the respiratory, digestive, and reproduction tracts (Figure 1). Fundamentally, the mucosal immune system has evolved to tolerate commensal microbes, while responding quickly and effectively to pathogenic challenges. Although the mucosal immune systems of mammals and birds share many features, which are fundamental to the functioning of mucosa-associated lymphoid tissues, the avian mucosal immune response has unique features. A fundamental difference between mammals and birds is the absence of encapsulated lymph nodes but the presence of diffuse lymphoid tissue in birds.

The mucosal tissue of the nose is the first to come into contact with particles and pathogens upon inhalation. In chickens, a major characteristic of the nasal-associated lymphoid tissue (NALT) is the formation of defined areas of B cells with caps composed of CD4^+^ T cells. Immunoglobulin-producing (Ig^+^) B cells are found both within the NALT structures and distributed throughout the epithelium, and are mostly IgY^+^ in chickens (1). In mammals, immunoglobulin class-switching from IgM to IgA occurs in the NALT and IgA-producing plasma cells are abundantly present in the nasal cavity. In chickens, closely related to the mucosal tissue of the nose are the head-associated lymphoid tissues, which include the Harderian gland and the conjunctiva-associated lymphoid tissue (CALT). The Harderian gland is located in the orbit of the eye and has the structure of a typical secondary lymphoid organ, with many B cells and plasma cells, germinal centers, and T cell-dependent interfollicular regions with scattered T cells and macrophages (2, 3). It plays an important role in the adaptive mucosal immune response upon ocular exposure to avian pathogens (4, 5). CALT is located on the inner surfaces of the eyelids and is observable in 1-week-old chickens (6). Based on the composition of CALT lymphocytes, the induction of antigen-specific IgA antibody-secreting cells after ocular exposure, the expansion of polymeric immunoglobulin receptors, and the production of IFNγ by lacrimal fluids suggests that CALT plays important roles in the avian mucosal immune response (7). It should be noted that most mammals (e.g., cats, dogs, and humans) also develop CALT. A recent study found that mice possess a similar lymphoid structure in the lacrimal sac, the so-called tear duct-associated lymphoid tissue, which plays an important role in the induction of an immune response by the ocular immunosurveillance system (8).

The chicken respiratory tract is very different from that of mammalians. For example, the avian lung has a unidirectional airflow (9) in contrast to the bidirectional airflow in the human lung. Furthermore, the bird lung is ventilated via air sacs, since birds do not have a diaphragm. A consequence of a unidirectional airflow is that particles are primarily deposited at the caudal regions of the lung (10), which is the part of the lung containing bronchus-associated lymphoid tissue (BALT). These highly organized lymphoid structures together with diffusely distributed cells were described for the first time in 1973 (11). Avian BALT structures are observed around 3–4 weeks post hatching and are fully developed in some birds at the age of 6 weeks (12). Both age and environmental stimuli influence BALT development (13). Furthermore, the number of BALT nodules increases significantly upon infection with pathogenic microorganisms (14). Another difference between the mammalian and chicken lung is the lack of alveolar macrophages at the surface of the air capillaries in chickens (14). Interestingly, a large network of macrophages and dendritic cells (DCs) is present in the mucosa of the larger airways, the linings of the parabronchi (15), and the connective tissue (9). Thus, phagocytic cells are strategically localized at the start of the gas-exchange areas to clear the air of inhaled particles before it reaches the thin and vulnerable air capillaries. Since chickens lack draining lymph nodes, the location where phagocytic cells present the particles to the immune system remains unclear. Presentation of particles may occur locally in the BALT, in the interstitial follicles between parabronchi, and/or in the spleen.

Gut-associated lymphoid tissues (GALTs) are well developed in birds (16). It consists of lymphoid cells located in the epithelial lining and the lamina propria as well as specialized lymphoid structures such as Peyer's patches and cecal tonsils. Peyer's patches in chicken are clearly visible at 2 weeks of age, and they increase in number with age. Like in mammals, they seem to consist of specialized epithelium with M cells that overlay structured follicles with defined T and B cell areas (17). Cecal tonsils, which are located in the neck region of each ceca, are structurally similar to Peyer's patches (18). Together, the GALT structures play an important role in the induction of immune responses (19).

## Mucosal vaccination in birds

Vaccination through the mucosa itself is frequently used in the poultry industry, as an economical, efficient, and reliable method to vaccinate large numbers of birds. However, a successful mucosal vaccine must elicit both local and systemic immune responses (20, 21). Poultry vaccines against viral infections consist of either live attenuated viruses or inactivated viruses formulated with a suitable adjuvant. Most live vaccines are applied mucosally via the oculo-nasal route or with a spray, so that the vaccine enters the respiratory tract or is taken up by the head-associated lymphoid tissues where it is recognized and taken up by antigen-presenting cells. The use of a spray is the preferred method for vaccination of birds against respiratory viruses, such as infectious bronchitis virus (22), Newcastle Disease virus, and avian metapneumovirus. However, although deposition patterns after aerosol or spray vaccination are conventionally studied using beads, the deposition pattern of beads is dependent on the bead size, the droplet size of the bead solution, and the age of the chickens. Larger beads (>3.7 μM) are mainly deposited in the upper respiratory tract, while smaller beads are distributed throughout the entire respiratory tract (23–25). The highest accumulation of beads occurs at the bifurcations primary to secondary bronchi (24), similar to that observed upon spray vaccination with avian influenza virus (AIV) (10), suggesting that particulate antigens are also taken up in the respiratory tract at these junctions. After entering the respiratory tract, particles are taken up by antigen-presenting cells (26) and are then presented to the immune system.

In addition to spray vaccination, vaccines can be delivered via drinking water. Vaccines dispensed through drinking water end up in the oral cavity with rapid transit to the esophagus and digestive tract. In this case, antigens will be taken up by cells in GALTs and presented to the immune system. Although oral vaccination has been reported to result in protection against *Salmonella* and reduction in necrotic enteritis lesions (27), other reports show less positive results (28). This may be related to the pathogen, type of vaccine, or age of the birds.

Inactivated vaccines are often poorly immunogenic and require additional components (adjuvants) for the induction of a protective immune response (29). These vaccines are formulated with a high antigenic mass of bacterial or viral origin conveyed in a suitable adjuvant, which renders these substances unsuitable for spray vaccination. Therefore, alternative strategies are needed for mucosal application of inactivated vaccines, such as specialized delivery systems or adjuvants with mucoadhesive properties. Several mucosal adjuvants have been employed in chickens and can be divided in two classes based on the mode of action: stimulation of the immune system and/or efficient delivery of vaccine materials. An important group of potential immune stimulators are the toll-like receptor (TLR)-based adjuvants (30). TLRs are pattern recognition receptors, a group of receptors present on immune cells that recognize the conserved molecular structures of pathogens, the so-called microbe-associated molecular patterns. The recognition of pathogens by TLRs results in the immediate activation of the immune system (31). CpG oligodeoxynucleotides (CpG ODNs), the ligand of chicken TLR21, have been reported as potential vaccine adjuvants in chickens. For example, vaccination with NDV and CpG resulted in the induction of specific immune responses and protection (32), and *in vivo* administration of CpG ODNs by itself suppressed the replication of IBV in the chicken embryo (33). Enhanced protection upon CpG ODN administration has also been reported for Marek's disease virus (34), as well as infection with *Salmonella enterica* (35) and *Escherichia coli* (36). Other potential immune stimulators include oligopeptides complexed with an agonistic anti-chicken CD40 monoclonal antibody (37) and the immune potentiator CVCVA5, which induces enhanced immune responses and protection against AIV upon vaccination (38, 39).

Mucoadhesive adjuvants, such as chitosan, have been suggested to increase the mucosal residence time, which results in increased antigen uptake and presentation (40). Rauw and colleagues investigated the effect of chitosan on the mucosal delivery of NDV vaccines in 1-day-old birds and found an enhanced cell mediated immunity in the spleen (41). Also, particulate deliverable systems, such as poly lactic-co-glycolic acid (PLGA) nanoparticles, invoke mechanisms that influence vaccine immunogenicity via enhanced antigen processing (42). Interestingly, vaccinating chickens with PLGA particles encapsulated with inactivated AIV vaccine adjuvanted with CpG ODNs resulted in enhanced antibody responses and a reduction in virus shedding (43). Furthermore, intranasal administration of NDV DNA vaccine-encapsulated nanoparticles in specific-pathogen-free chickens resulted in enhanced humoral and cellular immune responses and protection against challenge with a highly virulent NDV strain (44).

## Aspects of antigen delivery for mucosal vaccines

Approaches of mucosal vaccination, with delivery systems as developed for mammals, may turn out to be similarly effective in birds. In the case of mammals, it is well known that the function of Peyer's patches in the gut immune system is totally distinct from that of the lamina propria lymphoid tissues of intestinal villi (45). Fundamentally, antigen-specific intestinal immune responses to luminal substances are initiated in Peyer's patches, whereas the actual immune reactions (e.g., IgA production) take place in the intestinal villi (45). Therefore, DCs that prime mature naïve T cells by antigen presentation are frequently found in Peyer's patches; however, DCs are also abundantly distributed in the lamina propria (LP) of the gut intestinal villi, in mammals, despite the absence of lymphoid follicular structures, such as Peyer's patches (46). In birds, the presence of tissue DCs has not been well demonstrated due to the lack of specific antibodies. A first step was made by showing the presence of cells that express the C type lectin receptor DEC205^+^ in tissues, including bursa and spleen (47). Expression of chicken DEC205 reflects the unique structure and function of the avian immune system (47). In mammals, a subset of the LP DCs, which are monocyte-derived and express CX_3_CR1 (a receptor for CX_3_CL1), can access the intestinal lumen to directly sample luminal microorganisms by extending their dendrites to regulate immunological tolerance and inflammation (48). A recent study demonstrated that goblet cells, whose primary function is to produce mucus that covers intestinal epithelial surface, have an additional function to deliver luminal antigens to another subset of LP DCs that have differentiated from conventional myeloid DC precursors and express α_E_ integrin, known as CD103 (49). Among the two DC populations (i.e., CX_3_CR1^+^ DCs and CD103^+^ DCs) found in the LP of the gut, CD103^+^ DCs migrate into the mesenteric lymph nodes that drain the gastrointestinal tract to prime mature T cells for initiation of antigen-specific mucosal immune responses (50). Thus, a strategy that is capable of delivering the vaccine antigen to CD103^+^ DCs in the LP should be considered as a potential approach to increase the efficacy of mucosal vaccines (50). It should be noted, however, that chickens do not have mesenteric lymph nodes. Therefore, other routes for the delivery of antigens are present in chickens. Interestingly, it was demonstrated that a 12-mer peptide, which was discovered with the use of phage display technology, possesses broad targeting specificity for DCs of humans and mice (51). Moreover, the efficacy of orally administered lactic acid bacteria (LAB) that express the vaccine antigen together with the DC-specific peptide has been confirmed (52). Specifically, oral administration of DC-specific peptide-expressing LAB was shown to effectively induce antigen-specific immune responses in the gastrointestinal tract upon delivery to intestinal DCs (52). However, it is important to note that the mucosal tissues are lined by a tight epithelial barrier and also covered by a thick mucus layer (53). Moreover, CD103^+^ DCs present in the LP, which is located within intestinal tissues, are still far from the mucosal lumen in which the vaccine antigens are administered (46). Therefore, mucosal vaccines need to cross the physiological barrier (e.g., mucus layer and epithelial layer) to reach CD103^+^ DCs for initiation of intestinal immune responses. Although markers such as CD103^+^ are still lacking in birds, it seems reasonable to assume the presence of similar gut antigen-presenting cells in these species.

Another possible vaccine delivery system is with liposomes. More than 50 years ago, the British biophysicist Alec Bangham discovered spherical lipid bilayer structures, so-called liposomes, when testing a new electron microscope introduced in his research institution using dry phospholipid samples that were negatively stained (54). Liposomes are basically formed by phospholipids, which are composed of a hydrophilic head group linked to a hydrophobic tail by a glycerol backbone (55). The size of liposomes varies from small (nanoscale) to large (micro-scale) (55). A well-known biological characteristic of liposomes in vaccine development is the capability of enclosing several different biomaterials, such as protein antigens and nucleic adjuvants, regardless of solubility since liposomes possess amphiphilic features (55). The activity of liposomes can be freely modulated by chemical modification of the surface of the structure. For example, coating of liposomes with polyethylene glycol increases the retention effect in blood, compared with bare liposomes, because the coating allows the liposome to escape from capture by the reticuloendothelial system in the liver and spleen, etc. Another potential modification is to endow liposomes with tropism by conjugating cell-specific antibodies or potential ligand molecules that bind to specific receptors expressed by the target tissues or cells (56). Moreover, recent studies have succeeded in the development of heat-, pH-, enzyme-, and light-dependent liposomes as delivery vehicles that respond to certain stimuli *in vivo* (57). These liposomes have been also used for mucosal vaccine development (55). For example, cationic liposomes that are generated from cationic lipids, such as dimethyldioctadecylammonium bromide (58), 3β-[N-(N′,N′-dimethylaminoethane)-carbamoyl], and N-[1-(2,3-dioleoyloxy)propyl]-N,N,N-trimethylammonium methylsulfate (59), can be retained in the mucosal epithelium when administered via the mucosal route. To this end, the vaccine antigens and/or adjuvants enclosed in cationic liposomes are successfully released in the mucosal tissues, which results in immediate processing by DCs and subsequent induction of effective immune responses. Amphiphilic nanometer-sized gels, so-called nanogels, are effective biomaterials that can be used for not only drug delivery but also vaccine development (60). Pullulan, which is a polymer composed of regularly repeating glucose units, described as α(1-4)Glu-α(1-4)Glu-α(1-6)Glu, was first utilized to generate self-assembled nanogels by the addition of multihydrophobic domains consisting of 1.6 cholesteryl groups per 100 glucose units (61). One of the most attractive features of cholesterol-bearing pullulan (CHP) nanogels is the trapping of proteins in the nanoscale matrix, which contains a large amount of water (62, 63). So far, several bioactive proteins, including insulin (64), bovine serum albumin (62), α-chymotrypsin (65), and myoglobin (63), have been successfully encapsulated in CHP nanogels while maintaining activity. Similar to liposomes, the characteristics of CHP nanogels, such as electrical charge, can be freely altered by chemical modification (66). Recent studies of cationic CHP nanogels with encapsulation of several prototypes of vaccine antigens demonstrated that the antigen was effectively sampled by DCs present in the nasal mucosa and subsequent antigen-specific mucosal immune responses were effectively induced in not only mice, but also non-human primates, when administered intranasally (67–69). Because of the high potency of cationic CHP nanogel-based nasal vaccine, co-administration of a mucosal adjuvant is not required (67–69). Moreover, it should be noted that nasally administered cationic CHP nanogels and encapsulated vaccine antigens do not accumulate in the brain or olfactory bulb (67), suggesting that a strategy for nasal vaccine development using cationic CHP nanogels would be safe without the risk of undesired side effects, such as Bell's palsy (67).

## The possible negatively interfering effects of nutrients on mucosal vaccination

The mucosal immune system has exquisite qualities for maintaining immunological tolerance and the control of undesirable and counterproductive responses to nutrients. Therefore, successful mucosal vaccination would require overcoming mechanisms of mucosal tolerance. The vaccination effects of LAB illustrate the versatile characteristics of mucosal immune systems. However, intranasal administration of LAB, both live and killed, has been shown to produce an effective vaccination effect leading to protection against infection, oral administration was not effective. To reach any effect, frequent dosing for several weeks or novel delivery or adjuvant strategies was needed. Moreover, tolerization to antigens secreted by orally administered LAB has been reported (70).

Nutrients, such as the dietary antioxidant vitamin A, impact the tolerance of the mucosal immune system to a great extent. CD103^+^ DCs in the LP can convert vitamin A into retinoic acid, which, in combination with TGFβ, is one of the driving forces in the production of regulatory T cells (Tregs) in the mesenteric lymph nodes (71). Interestingly, vitamin A supplementation during lactation was shown to reduce allergic sensitization in the offspring of mice. Furthermore, through different mechanisms, dietary supplementation with probiotics, prebiotics, and n-3 polyunsaturated fatty acids, is suggested to support oral tolerance and to prevent allergy in early childhood (72). Also, in combination with certain members of the gut microbiota, nutrients are known to promote tolerance. Fermentation of non-digestible dietary carbohydrates (fibers) by the gut microbiota leads to production of short-chain fatty acids, such as acetate, propionate, and butyrate, which also have the capacity to stimulate the expansion and immuno-suppressive capacity of Tregs in the gut (73). L-arginine promotes lymphocyte proliferation, balances pro-inflammatory (IFN-γ and IL-2) and anti-inflammatory (IL-4 and IL-10) cytokines, and increases the secretory IgA (sIgA) level in burn-injured mice (74). In this regard, L-arginine supplementation inhibits *Clostridium perfringens* overgrowth and alleviates intestinal mucosal injury by modulating innate immune responses in chickens by enhancing barrier function and producing NO (75). Another study also suggests that L-arginine supplementation attenuates intestinal mucosal disruption in coccidiosis-challenged chickens probably through suppressing TLR4 and activating mTOR complex 1 pathways (76). Probiotic feeding is also appropriate to manipulate mucosal immunity. After 21 days of treatment with *Lactobacillus acidophilus* as a probiotic on T cells in chicken, the percentages of blood CD4^+^, CD8^+^, and TCR1^+^ cells were significantly higher in the probiotic-fed group than in the control group. After 14 days of the probiotic, a significantly greater number of CD4^+^ T cells were found in the ileum of probiotic-fed chickens, and this difference was even greater after 21 days. The findings indicated that probiotics may alter the distribution of T cells in the blood and lymphoid tissues in young chickens; however, transient changes in lymphoid tissues, indicating that probiotics likely do not permanently affect mucosal immunity (77). The effects of *Saccharomyces boulardii* and *Bacillus subtilis* on cytokine expression responses via Toll-like receptors (TLRs) by intestinal epithelial cells were to decrease the expression levels of INF-γ and IL-8 and to increase the levels of serum IgA and sIgA in mucosa (78). These results indicated that *Saccharomyces boulardii* and *Bacillus subtilis* have a role in inducing mucosal innate immunity in chickens (78).

Some dietary products have the capacity to co-induce stress proteins in gut-associated cells. Oral administration of carvacrol, essential oil of oregano, was found to inhibit experimental autoimmune arthritis in mice. Upon further analysis, this compound actually co-induced the expression of heat shock protein 70 (HSP70) in cells in Peyer's patches. Subsequently, the enhanced HSP70 expression in PPs led to the activation and expansion of HSP70-specific T cells with regulatory, IL-10-producing capacities (79). Taken together, these findings suggest that dietary components may promote tolerance by various underlying mechanisms. However, the identity of such dietary factors that impact the efficacy of mucosal vaccination remains to be further elucidated.

## Conclusion

Immune protection against infection is considered to be the most efficient when localized at the sites of entry of the infectious agent. Since most infections occur at mucosal surfaces, mucosal vaccination is an actively sought research goal in many species, including mammals and birds. Despite successes, such as with polio and rotavirus vaccination in humans, the scientific challenges in this area are still manifold. And this is, partly, due to the fact that oral administration of proteins induces tolerance, and not immune activation. Given the existing similarities between the mucosal immune systems of mammals and birds, it is possible to effectively use in birds some of the successful mucosal vaccination strategies as developed for mammals.

## Author contributions

All authors listed have made a substantial, direct and intellectual contribution to the work, and approved it for publication.

### Conflict of interest statement

The authors declare that the research was conducted in the absence of any commercial or financial relationships that could be construed as a potential conflict of interest.

## References

[1] Ohshima K Hiramatsu K. Distribution of T-cell subsets and immunoglobulin-containing cells in nasal-associated lymphoid tissue (NALT) of chickens. Histol Histopathol. (2000) 15:713–20. 10.14670/HH-15.713 10963115

[2] Jeurissen SH Janse EM Koch G De Boer GF. Postnatal development of mucosa-associated lymphoid tissues in chickens. Cell Tissue Res. (1989) 258:119–24. 2805039 10.1007/BF00223151

[3] Savage ML Olah I Scott TR. Plasma cell proliferation in the chicken harderian gland. Cell Prolif. (1992) 25:337–44. 1643190 10.1111/j.1365-2184.1992.tb01444.x

[4] Burns RB. Specific antibody production against a soluble antigen in the Harderian gland of the domestic fowl. Clin Exp Immunol. (1976) 26:371–4. 791553 PMC1540858

[5] van Ginkel FW van Santen VL Gulley SL Toro H. Infectious bronchitis virus in the chicken Harderian gland and lachrymal fluid: viral load, infectivity, immune cell responses, and effects of viral immunodeficiency. Avian Dis. (2008) 52:608–17. 10.1637/8349-050908-Reg.1 19166051

[6] Fix AS Arp LH. Quantification of particle uptake by conjunctiva-associated lymphoid tissue (CALT) in chickens. Avian Dis. (1991) 35:174–9. 2029252

[7] van Ginkel FW Gulley SL Lammers A Hoerr FJ Gurjar R Toro H. Conjunctiva-associated lymphoid tissue in avian mucosal immunity. Dev Comp Immunol. (2012) 36:289–97. 10.1016/j.dci.2011.04.012S0145-305X(11)00127-3 21641931

[8] Nagatake T Fukuyama S Kim DY Goda K Igarashi O Sato S. Id2-, RORgammat-, and LTbetaR-independent initiation of lymphoid organogenesis in ocular immunity. J Exp Med. (2009) 206:2351–64. 10.1084/jem.20091436jem.20091436 19822644 PMC2768868

[9] Reese S Dalamani G Kaspers B. The avian lung-associated immune system: a review. Vet Res. (2006) 37:311–24. 10.1051/vetres:2006003v6021 16611550

[10] Reemers SS van Haarlem DA Groot Koerkamp MJ Vervelde L. Differential gene-expression and host-response profiles against avian influenza virus within the chicken lung due to anatomy and airflow. J Gen Virol. (2009) 90:2134–46. 10.1099/vir.0.012401-0vir.0.012401-0 19494054

[11] Bienenstock J Johnston N Perey DY. Bronchial lymphoid tissue. I. Morphologic characteristics. Lab Invest. (1973) 28:686–92. 4123478

[12] Fagerland JA Arp LH. Structure and development of bronchus-associated lymphoid tissue in conventionally reared broiler chickens. Avian Dis. (1993) 37:10–18. 8452486

[13] Jeurissen SHM Vervelde L Janse EM Structure and function of lymphoid tissues of the chicken. Poultry Sci Rev. (1994) 5:183–207.

[14] Van Alstine WG Arp LH. Histologic evaluation of lung and bronchus-associated lymphoid tissue in young turkeys infected with Bordetella avium. Am J Vet Res. (1988) 49:835–39. 3400920

[15] Maina JN. Development, structure, and function of a novel respiratory organ, the lung-air sac system of birds: to go where no other vertebrate has gone. Biol Rev Camb Philos Soc. (2006) 81:545–79. 10.1017/S1464793106007111 17038201

[16] Casteleyn C Doom M Lambrechts E Van den Broeck W Simoens P Cornillie P. Locations of gut-associated lymphoid tissue in the 3-month-old chicken: a review. Avian Pathol. (2010) 39:143–50. 10.1080/03079451003786105922993774 20544418

[17] Befus AD Johnston N Leslie GA Bienenstock J. Gut-associated lymphoid tissue in the chicken. I. Morphology, ontogeny, and some functional characteristics of Peyer's patches. J Immunol. (1980) 125:2626–32. 7430642

[18] Kitagawa H Hiratsuka Y Imagawa T Uehara M. Distribution of lymphoid tissue in the caecal mucosa of chickens. J Anat.(1998) 192(Pt. 2): 293–8. 9643430 10.1046/j.1469-7580.1998.19220293.x PMC1467763

[19] Bar-Shira E Sklan D Friedman A. Establishment of immune competence in the avian GALT during the immediate post-hatch period. Dev Comp Immunol. (2003) 27:147–57. 12543128 10.1016/s0145-305x(02)00076-9

[20] Ryan EJ Daly LM Mills KH. Immunomodulators and delivery systems for vaccination by mucosal routes. Trends Biotechnol. (2001) 19:293–304. 11451471 10.1016/s0167-7799(01)01670-5

[21] van Ginkel FW Nguyen HH McGhee JR. Vaccines for mucosal immunity to combat emerging infectious diseases. Emerg Infect Dis. (2000) 6:123–32. 10.3201/eid0602.000204 10756145 PMC2640846

[22] De Wit JJ Swart WA Fabri TH. Efficacy of infectious bronchitis virus vaccinations in the field: association between the alpha-IBV IgM response, protection and vaccine application parameters. Avian Pathol. (2010) 39:123–31. 10.1080/03079451003604639921016407 20390547

[23] Hayter RB Besch EL. Airborne-particle deposition in the respiratory tract of chickens. Poult Sci. (1974) 53:1507–11. 10.3382/ps.0531507 4850471

[24] Tell LA Smiley-Jewell S Hinds D Stephens KE Teague SV Plopper CG. An aerosolized fluorescent microsphere technique for evaluating particle deposition in the avian respiratory tract. Avian Dis. (2006) 50:238–44. 10.1637/7427-082605R.1 16863074

[25] Corbanie EA Matthijs MG van Eck JH Remon JP Landman WJ Vervaet C. Deposition of differently sized airborne microspheres in the respiratory tract of chickens. Avian Pathol. (2006) 35:475–85. 10.1080/03079450601028845 17121737

[26] de Geus ED Jansen CA Vervelde L Uptake of particulate antigens in a nonmammalian lung: phenotypic and functional characterization of avian respiratory phagocytes using bacterial or viral antigens. J Immunol. (2012) 188:4516–26. 10.4049/jimmunol.1200092jimmunol.1200092 22467653

[27] Eeckhaut V Haesebrouck F Ducatelle R Van Immerseel F. Oral vaccination with a live *Salmonella Enteritidis/*Typhimurium bivalent vaccine in layers induces cross-protection against caecal and internal organ colonization by a Salmonella Infantis strain. Vet Microbiol. (2018) 218:7–12. 10.1016/j.vetmic.2018.03.022 29685223

[28] Leigh SA Evans JD Collier SD Branton SL. The impact of vaccination route on *Mycoplasma gallisepticum* vaccine efficacy. Poult Sci. (2018) 10.3382/ps/pey1884999283 [Epub ahead of print]. 29788205

[29] Meeusen EN Walker J Peters A Pastoret PP Jungersen G. Current status of veterinary vaccines. Clin Microbiol Rev. (2007) 20:489–510. 10.1128/CMR.00005-07 17630337 PMC1932753

[30] Gupta SK Deb R Dey S Chellappa MM. Toll-like receptor-based adjuvants: enhancing the immune response to vaccines against infectious diseases of chicken. Expert Rev Vaccines (2014) 13:909–25. 10.1586/14760584.2014.920236 24855906

[31] Akira S Takeda K Kaisho T. Toll-like receptors: critical proteins linking innate and acquired immunity. Nat Immunol. (2001) 2:675–80. 10.1038/9060990609 11477402

[32] Linghua Z Xingshan T Fengzhen Z. Vaccination with Newcastle disease vaccine and CpG oligodeoxynucleotides induces specific immunity and protection against Newcastle disease virus in SPF chicken. Vet Immunol Immunopathol. (2007) 115:216–22. 10.1016/j.vetimm.2006.10.017 17157392

[33] Dar A Potter A Tikoo S Gerdts V Lai K Babiuk LA. CpG oligodeoxynucleotides activate innate immune response that suppresses infectious bronchitis virus replication in chicken embryos. Avian Dis. (2009) 53:261–7. 10.1637/8560-121808-Reg.1 19630234

[34] Parvizi P Mallick AI Haq K Haghighi HR Orouji S Thanthrige-Don N. A toll-like receptor 3 ligand enhances protective effects of vaccination against Marek's disease virus and hinders tumor development in chickens. Viral Immunol. (2012) 25:394–401. 10.1089/vim.2012.0033 22857262

[35] He H Genovese KJ Swaggerty CL Nisbet DJ Kogut MH. *In vivo* priming heterophil innate immune functions and increasing resistance to Salmonella enteritidis infection in neonatal chickens by immune stimulatory CpG oligodeoxynucleotides. Vet Immunol Immunopathol. (2007) 117:275–83. 10.1016/j.vetimm.2007.03.002 17434210

[36] Gomis S Babiuk L Allan B Willson P Waters E Hecker R. Protection of chickens against a lethal challenge of Escherichia coli by a vaccine containing CpG oligodeoxynucleotides as an adjuvant. Avian Dis. (2007) 51:78–83. 10.1637/0005-2086(2007).051[0078:POCAAL]2.0.CO;2 17461270

[37] Chou WK Chen CH Vuong CN Abi-Ghanem D Waghela SD Mwangi W. Significant mucosal sIgA production after a single oral or parenteral administration using *in vivo* CD40 targeting in the chicken. Res Vet Sci. (2016) 108:112–5. 10.1016/j.rvsc.2016.08.013S0034-5288(16)30257-0 27663378

[38] Tang Y Lu J Wu P Liu Z Tian Z Zha G. Inactivated vaccine with adjuvants consisting of pattern recognition receptor agonists confers protection against avian influenza viruses in chickens. Vet Microbiol. (2014) 172:120–8. 10.1016/j.vetmic.2014.05.007S0378-1135(14)00243-0 24894132 PMC4208718

[39] Lu J Wu P Zhang X Feng L Dong B Chu X. Immunopotentiators improve the efficacy of oil-emulsion-inactivated avian influenza vaccine in chickens, ducks and geese. PLoS ONE (2016) 11:e0156573. 10.1371/journal.pone.0156573PONE-D-15-27158 27232188 PMC4883754

[40] O'Hagan DT Valiante NM. Recent advances in the discovery and delivery of vaccine adjuvants. Nat Rev Drug Discov. (2003) 2:727–35. 10.1038/nrd1176nrd1176 12951579 PMC7097252

[41] Rauw F Gardin Y Palya V Anbari S Gonze M Lemaire S. The positive adjuvant effect of chitosan on antigen-specific cell-mediated immunity after chickens vaccination with live Newcastle disease vaccine. Vet Immunol Immunopathol. (2010) 134:249–58. 10.1016/j.vetimm.2009.10.028S0165-2427(09)00354-7 19939464

[42] Men Y Audran R Thomasin C Eberl G Demotz S Merkle HP. MHC class I- and class II-restricted processing and presentation of microencapsulated antigens. Vaccine (1999) 17:1047–56. 10195614 10.1016/s0264-410x(98)00321-1

[43] Singh SM Alkie TN Nagy E Kulkarni RR Hodgins DC Sharif S. Delivery of an inactivated avian influenza virus vaccine adjuvanted with poly(D,L-lactic-co-glycolic acid) encapsulated CpG ODN induces protective immune responses in chickens. Vaccine (2016) 34:4807–13. 10.1016/j.vaccine.2016.08.009S0264-410X(16)30666-1 27543454

[44] Zhao K Rong G Hao Y Yu L Kang H Wang X. IgA response and protection following nasal vaccination of chickens with Newcastle disease virus DNA vaccine nanoencapsulated with Ag@SiO2 hollow nanoparticles. Sci Rep. (2016) 6:25720. 10.1038/srep25720srep25720 27170532 PMC4864420

[45] Neutra MR Kozlowski PA. Mucosal vaccines: the promise and the challenge. Nat Rev Immunol. (2006) 6:148–58. 10.1038/nri1777 16491139

[46] Schiavi E Smolinska S O'Mahony L. Intestinal dendritic cells. Curr Opin Gastroenterol. (2015) 31:98–103. 10.1097/MOG.000000000000015500001574-201503000-00004 25651073

[47] Staines K Young JR Butter C. Expression of chicken DEC205 reflects the unique structure and function of the avian immune system. PLoS ONE (2013) 8:e51799. 10.1371/journal.pone.0051799PONE-D-11-21674 23326318 PMC3541370

[48] Niess JH Brand S Gu X Landsman L Jung S McCormick BA. CX3CR1-mediated dendritic cell access to the intestinal lumen and bacterial clearance. Science (2005) 307:254–8. 10.1126/science.1102901 15653504

[49] McDole JR Wheeler LW McDonald KG Wang B Konjufca V Knoop KA. Goblet cells deliver luminal antigen to CD103+ dendritic cells in the small intestine. Nature (2012) 483:345–9. 10.1038/nature10863nature10863 22422267 PMC3313460

[50] Owen JL Sahay B Mohamadzadeh M. New generation of oral mucosal vaccines targeting dendritic cells. Curr Opin Chem Biol. (2013) 17:918–24. 10.1016/j.cbpa.2013.06.013S1367-5931(13)00115-4 23835515 PMC3855095

[51] Curiel TJ Morris C Brumlik M Landry SJ Finstad K Nelson A. Peptides identified through phage display direct immunogenic antigen to dendritic cells. J Immunol. (2004) 172:7425–31. 10.4049/jimmunol.172.12.7425 15187120

[52] Mohamadzadeh M Durmaz E Zadeh M Pakanati KC Gramarossa M Cohran V. Targeted expression of anthrax protective antigen by Lactobacillus gasseri as an anthrax vaccine. Future Microbiol. (2010) 5:1289–96. 10.2217/fmb.10.78 20722604

[53] Nochi T Kiyono H. Innate immunity in the mucosal immune system. Curr Pharm Des. (2006) 12:4203–13. 17100623 10.2174/138161206778743457

[54] Bangham AD Standish MM Watkins JC. Diffusion of univalent ions across the lamellae of swollen phospholipids. J Mol Biol. (1965) 13:238–52. 5859039 10.1016/s0022-2836(65)80093-6

[55] Bernasconi V Norling K Bally M Hook F Lycke NY. Mucosal vaccine development based on liposome technology. J Immunol. Res. (2016) 2016:5482087. 10.1155/2016/5482087 28127567 PMC5227169

[56] Suk JS Xu Q Kim N Hanes J Ensign LM. PEGylation as a strategy for improving nanoparticle-based drug and gene delivery. Adv Drug Deliv Rev. (2016) 99:28–51. 10.1016/j.addr.2015.09.012 26456916 PMC4798869

[57] Mura S Nicolas J Couvreur P. Stimuli-responsive nanocarriers for drug delivery. Nat. Mater (2013) 12:991–1003. 10.1038/nmat3776nmat3776 24150417

[58] Ghaffar KA Marasini N Giddam AK Batzloff MR Good MF Skwarczynski M. Liposome-based intranasal delivery of lipopeptide vaccine candidates against group A streptococcus. Acta Biomater. (2016) 41:161–8. 10.1016/j.actbio.2016.04.012S1742-7061(16)30165-9 27063491

[59] Tada R Muto S Iwata T Hidaka A Kiyono H Kunisawa J. Attachment of class B CpG ODN onto DOTAP/DC-chol liposome in nasal vaccine formulations augments antigen-specific immune responses in mice. BMC Res Notes (2017) 10:68. 10.1186/s13104-017-2380-810.1186/s13104-017-2380-8 28126014 PMC5270218

[60] Terech P Weiss RG. Low molecular mass gelators of organic liquids and the properties of their gels. Chem Rev. (1997) 97:3133–60. 11851487 10.1021/cr9700282

[61] Akiyoshi K Deguchi S Moriguchi N Yamaguchi S Sunamoto J Self-aggregates of hydrophobized polysaccharides in water. Formation and characteristics of nanoparticles. Macromolecules (1993) 26:3062–8. 10.1021/ma00064a011

[62] Nishikawa T Akiyoshi K Sunamoto J Macromolecular complexation between bovine serum albumin and the self-assembled hydrogel nanoparticle of hydrophobized polysaccharides. J Am Chem Soc. (1996) 118:6110–5. 10.1021/ja953843c

[63] Akiyoshi K Nagai K Nishikawa T Sunamoto J Self-aggregates of Hydrophobized Polysaccharide as a Host for Macromolecular Guests. Chem Lett. (1992) 21:1727–30. 10.1158/1078-0432.CCR-06-1546

[64] Akiyoshi K Kobayashi S Shichibe S Mix D Baudys M Kim SW. Self-assembled hydrogel nanoparticle of cholesterol-bearing pullulan as a carrier of protein drugs: complexation and stabilization of insulin. J Control Release (1998) 54:313–20. 9766251 10.1016/s0168-3659(98)00017-0

[65] Nishikawa T Akiyoshi K Sunamoto J Supramolecular assembly between nanoparticles of hydrophobized polysaccharide and soluble protein complexation between the self-aggregate of cholesterol-bearing pullulan and alpha-chymotrypsin. Macromolecules (1994) 27:7654–9. 10.1006/jmbi.1998.1881

[66] Ayame H Morimoto N Akiyoshi K. Self-assembled cationic nanogels for intracellular protein delivery. Bioconjug Chem. (2008) 19:882–90. 10.1021/bc700422s 18336000

[67] Nochi T Yuki Y Takahashi H Sawada S Mejima M Kohda T. Nanogel antigenic protein-delivery system for adjuvant-free intranasal vaccines. Nat Mater. (2010) 9:572–8. 10.1038/nmat2784nmat2784 20562880

[68] Kong IG Sato A Yuki Y Nochi T Takahashi H Sawada S. Nanogel-based PspA intranasal vaccine prevents invasive disease and nasal colonization by *Streptococcus pneumoniae*. Infect Immun. (2013) 81:1625–34. 10.1128/IAI.00240-13IAI.00240-13 23460513 PMC3647999

[69] Fukuyama Y Yuki Y Katakai Y Harada N Takahashi H Takeda S. Nanogel-based pneumococcal surface protein A nasal vaccine induces microRNA-associated Th17 cell responses with neutralizing antibodies against Streptococcus pneumoniae in macaques. Mucosal Immunol. (2015) 8:1144–53. 10.1038/mi.2015.5mi20155 25669148 PMC4762909

[70] Wells J. Mucosal vaccination and therapy with genetically modified lactic acid bacteria. Annu Rev Food Sci Technol. (2011) 2:423–45. 10.1146/annurev-food-022510-133640 22129390

[71] Coombes JL Siddiqui KR Arancibia-Carcamo CV Hall J Sun CM Belkaid Y. A functionally specialized population of mucosal CD103+ DCs induces Foxp3+ regulatory T cells via a TGF-beta and retinoic acid-dependent mechanism. J Exp Med. (2007) 204:1757–64. 10.1084/jem.20070590 17620361 PMC2118683

[72] Kostadinova AI Pablos-Tanarro A Diks MAP van Esch B Garssen J Knippels LMJ. Dietary intervention with beta-lactoglobulin-derived peptides and a specific mixture of fructo-oligosaccharides and bifidobacterium breve M-16V facilitates the prevention of whey-induced allergy in mice by supporting a tolerance-prone immune environment. Front Immunol. (2017) 8:1303. 10.3389/fimmu.2017.01303 29123515 PMC5662887

[73] Bollrath J Powrie F. Immunology. feed your tregs more fiber. Science (2013) 341:463–4. 10.1126/science.1242674341/6145/463 23908210

[74] Fan J Meng Q Guo G Xie Y Li X Xiu Y. (2010). Effects of early enteral nutrition supplemented with arginine on intestinal mucosal immunity in severely burned mice. Clin. Nutr. (2013) 29:124–30. 10.1016/j.clnu.2009.07.005 19783080

[75] Zhang B Lv Z Li H Guo S Liu D Guo Y. Dietary l-arginine inhibits intestinal Clostridium perfringens colonisation and attenuates intestinal mucosal injury in broiler chickens. Br J. Nutr. (2017) 118:321–32. 10.1017/S0007114517002094 28901890

[76] Tan J Applegate TJ Liu S Guo Y Eicher SD. Supplemental dietary L-arginine attenuates intestinal mucosal disruption during a coccidial vaccine challenge in broiler chickens. Br J Nutr. (2014) 112:1098–109. 10.1017/S0007114514001846S0007114514001846 25181320

[77] Asgari F Madjd Z Falak R Bahar MA Nasrabadi MH Raiani M. Probiotic feeding affects T cell populations in blood and lymphoid organs in chickens. Benef Microbes (2016) 7:669–75. 10.3920/BM2016.0014 27349931

[78] Rajput IR Ying H Yajing S Arain MA Weifen L Ping L *Saccharomyces boulardii* and *Bacillus subtilis B10* modulate TLRs and cytokines expression patterns in jejunum and ileum of broilers. PLoS ONE (2017) 12:e0173917 10.1371/journal.pone.0173917PONE-D-16-49094 28319123 PMC5358784

[79] Wieten L van der Zee R Spiering R Wagenaar-Hilbers J van Kooten P Broere F. A novel heat-shock protein coinducer boosts stress protein Hsp70 to activate T cell regulation of inflammation in autoimmune arthritis. Arthritis Rheum. (2010). 62:1026–35. 10.1002/art.27344 20131272

